# New Use of Cocaine

**Published:** 1889-04

**Authors:** L. G. Hardman

**Affiliations:** Harmony Grove, Ga.


### NEW USE OF COCAINE.

BY L. G. HARDMAN, M. D., HARMONY GROVE, GA.

Knowing that surgeons are often called on in various sections of the country to do operations on the horse, and one of the greatest hindrances to these operations is to secure the animal so the operator will not be constantly interrupted in his work; and to overcome this difficulty I have been using a 5 per cent. solution of muriate of cocaine, injected at the point of incision, which destroys the sensibility completely, so the animal will not even move his leg or any other part, and does not show any signs of pain whatever. There is some risk in throwing the horse in order to secure him; and further, does him some damage in tying him down or securing in stocks, and this overcomes all of these difficulties. The solution should be injected under the skin all around where you expect to cut, and even in the tumor. I have removed a tumor of considerable size from the anterior surface of the front leg of a horse without any indication of pain whatever. I do not know whether an anæsthetic could be administered to a horse without much danger or not, but it is seldom done. This solution, or even stronger, could be used with safety. For small tumors, such as warty growth, the injection need not be introduced anywhere except at the base. This is also true of all pedunculated tumors. It can be used also in the other animals, such as the dog, although they can be anæsthetized with much less trouble and risk.

I do not know that the cocaine has been used this way before in animals, but I have been using it for some time in the human for the removal of tumors, but this part is not all original with me; but the use in the horse is, so far as I know.

